# Prevention and control of serious trauma and accidental injury in China: Timely but difficult

**DOI:** 10.4103/2321-3868.113327

**Published:** 2015-06-26

**Authors:** Xiaobing Fu, Shuliang Lu, Zhengguo Wang

**Affiliations:** 1 Burn Institute, The Ruijin Hospital, Shanghai Jiaotong University, Shanghai; 2 The Institute of Surgery, The Third Military Medical University, Chongqing, P. R. China; 3 Wound Healing Unit, The College of Life Science, The PLA General Hospital, Beijing, 100853; 4 Wound Healing Unit, the First Affiliated Hospital, Chinese PLA General Hospital, Trauma Center of Postgraduate Medical School, 51 Fu Cheng Road, Beijing, 100048 P. R. China

**Keywords:** Trauma, accidental injury, prevention, control

## Abstract

Serious trauma and accidental injury are the leading causes of death among people younger than 45 years old in China. Thus, the prevention and control of serious trauma and accidental injury are important for reducing these deaths. The concept is timely but difficult. Here, we review the current state of serious trauma and accidents in China and other countries, focusing on road accidents, and provide our personal perspectives and suggestions on how to prevent and control these serious injuries in China.

Trauma and accidental injury mainly refer to injuries and deaths caused by road accidents, fires, mining disasters, food poisoning and other emergencies. Trauma and accidental injuries have become the fourth largest cause of injuries and deaths in China in general and the first cause among people younger than 45 years old. Trauma and accidental injuries have become a main threat to health and life, lowering the quality of life and work and decimating the labor force, which both influence the development and stability of our society. Therefore, the prevention and control of trauma and accidental injury are important strategies for the nation and essential measures in responding to changes in the public-health arena. All sectors should pay attention and take proactive measures to ensure the prevention and control of trauma.

**Table 1:** 

Access this article online	
**Quick Response Code**:	**Website**: www.burnstrauma.com
	**DOI**: 10.4103/2321-3868.113327

Road accidents have cost nearly 1.3 million lives in China (67,759 people in 2010) and have resulted in injuries to 50 million people (275,125 in 2010). These accidents are among the top three causes of death among people 5 to 44 years old (and the number-one cause of death among people 15 to 29 years old). Road accidents will cause the death of an estimated 2.4 million people by 2020.[1] Currently, the economic loss caused by road accidents is an estimated 1–3% of the global gross domestic product, or more than US$ 500 billion. To address this growing issue, the United Nations Assembly passed Resolutions 58/289 and A/RES/64/255, requiring common efforts from all countries and regions to stabilize and lower the death rate caused by road accidents.[2]

In 2011, a major railway accident that occurred on the Ningbo-Wenzhou high-speed railway caused 40 deaths and 172 injuries, and a school bus accident that occurred in Yulinzi Town, Gansu Province, resulted in 21 deaths. Such horrifying cases have attracted people’s attention to road accidents and resulted in discussions to reform the school bus system. We lack detailed statistics on injures caused by fire, but experts estimate that 5 to 10 million people suffer burn injuries or die of burns each year in China. Although China has the highest rate of successfully treating burns in the world, disability caused by severe burns still prevents a large number of people from working and having normal social activities, which has added to the economic burden on our social system.

Among natural disasters and major mine accidents, the damage from the massive 2008 earthquake in Wenchuan County, Sichuan Province, is still fresh in people’s memories. The earthquake caused 69,134 deaths, 374,061 injuries and 17,681 missing people, resulting in 46,162,165 affected people. The direct economic loss reached RMB 845.1 billion.[3,4] After the earthquake, studies revealed that certain people died or became disabled because of inadequate first-aid treatment.

According to the World Health Organization, 20% of people involved in road accidents die due to delayed first-aid, and two-thirds of such deaths occur within 25 min after the trauma. In China, 60% of such deaths occur before patients arrive at the hospital. Therefore, road aid and rescue must be timely and immediate, and concepts such as the “gold 10 minutes”, the “golden hour” and “first-aid starts in the field” have become widely accepted.

Trauma and accidental injury can cause tremendous damage and lead to horrifying consequences for people’s lives and property. Sudden disasters have been instructive, and advanced countries have taken action to teach the public basic first-aid knowledge through seminars, workshops, newspapers, magazines, booklets and the internet. The penetration rate of first-aid knowledge has reached 40% in France and 60% in Germany. In the US, people have a strong awareness about administering first-aid, and the penetration rate of relevant information is as high as 89.95%, with the number of people receiving basic first-aid training exceeding 70 million, or about one-third of the population. Several countries have included first-aid techniques in their basic education curricula. In Germany, children younger than 10 years old must receive 8 hours of first-aid training, and adolescents between 10 and 16 years are required to take a course for 1.5 days. Additionally, strict laws ensure that specialized industry workers receive regulated first-aid training. For example, patrol officers in Italy must undergo basic first-aid training, and US firemen, police and security guards must receive training for at least 21 hours. Drivers of school buses and coaches in certain US states must pass basic life-support first-aid training.

In addition, people who administer first-aid without asking for a reward should be protected by law, such as the Good Samaritan Law, which has been passed by most US state governments.[5] Such people are mainly non-professionals who provide the first level of aid (first responders). The Good Samaritan Law encourages people to aid each other in emergencies. The law calls for people to use their common sense under such circumstances and to try their best to save lives and reduce deaths and injuries based on ability, knowledge and skills.

China is taking active measures to prevent and control trauma and accidental injury. A series of actions has been initiated to promote and educate the public, implementing mandatory management measures (such as banning drunk driving) and developing emergency system and treatment techniques, such as setting up pre-hospital emergency reaction chains and emergency hotlines and organizing promotion and training through the Red Cross. However, in general, people are still ignorant about the severity of trauma and accidental injury and first-aid knowledge and skills. We need large-scale promotion and education in this area and national strategies to prevent and control such accidents. In the current environment, in many places, the emergency response is relatively slow, and professional first-aiders cannot reach the accident field in time. Additionally, people nearby, including policemen, firemen and other drivers, lack basic first-aid knowledge and cannot offer the necessary help, which results in delay in first-aid and medical treatment and preventable deaths. In several recent cases, people administering first-aid were wrongly accused of being responsible for the accident. This situation has cultivated worries about legal issues in such cases. An increasing awareness of the law has stopped people from helping others, as in the Nanjing Pengyu incident, in which a toddler was run over and not helped by passersby.

We propose the following strategies to prevent and reduce injury and to protect first responders. First, we must establish a particular day for trauma and accidental injury promotion, recruiting medical practitioners in the areas of burns, severe diseases, first-aid and medical management; traffic police and Red Cross workers to give lectures on the significance of trauma and accidental injury, early perception and first-aid skills. Such a day would inform the public that trauma and accidental injury are preventable, increase public awareness and encourage the public to take action. Another goal should be to teach the public how to administer basic first-aid, such as judging the state of the injured person, calling for help, putting the person in the right body position and helping the person leave the scene of the accident if possible. Such activities can help to prevent and control trauma and accidental injury at a national level. Second, we must provide personnel in specialized occupations with compulsory first-aid training before these individuals start working or acquire professional certification. These occupations include the military, police, firemen, security guards, road administrators, tour guides and drivers. With the help of simple tools, these professionals can administer basic first-aid, including cardiopulmonary resuscitation, aeration, wound wrapping and homeostasis, simple immobilization and transportation. In doing so, these professionals could provide first-aid while waiting for professional first-aiders, reducing the risk of disability and complications, and saving lives. Third, we must establish a special fund for preventing and controlling trauma and accidental injury and construct a comprehensive prevention system. In case of an unexpected, large-scale accident, strong material backup and stable funding are essential. Such funding is important for corresponding education and training. Fourth, we must conduct data collection for and monitoring and policy review of trauma and accidental injury. We need to thoroughly review the relevant policies and regulations in China and the rest of the world to lay a solid theoretical foundation for establishing a suitable prevention and control system in China. Fifth, we must consider drafting regulations and taking legislative measures to protect people offering first-aid (including medical aid and other necessary measures to protect injured people) in emergencies, without any associated aspirations for gain.
